# Immunization with *Toxoplasma gondii GRA17* Deletion Mutant Induces Partial Protection and Survival in Challenged Mice

**DOI:** 10.3389/fimmu.2017.00730

**Published:** 2017-06-29

**Authors:** Jin-Lei Wang, Hany M. Elsheikha, Wei-Ning Zhu, Kai Chen, Ting-Ting Li, Dong-Mei Yue, Xiao-Xuan Zhang, Si-Yang Huang, Xing-Quan Zhu

**Affiliations:** ^1^State Key Laboratory of Veterinary Etiological Biology, Key Laboratory of Veterinary Parasitology of Gansu Province, Lanzhou Veterinary Research Institute, Chinese Academy of Agricultural Sciences, Lanzhou, China; ^2^Faculty of Medicine and Health Sciences, School of Veterinary Medicine and Science, University of Nottingham, Loughborough, United Kingdom; ^3^College of Animal Science and Veterinary Medicine, Shandong Agricultural University, Taian, China

**Keywords:** *Toxoplasma gondii*, immunization, live-attenuated vaccine, Δ*GRA17*, Th1/Th2 cytokines

## Abstract

Toxoplasmosis remains a world-threatening disease largely because of the lack of a fully effective vaccine. Here, we created a Δ*GRA17* mutant by disrupting the virulence factor *GRA17* using CRISPR-Cas9 method. Then, we tested whether Δ*GRA17* tachyzoites can be used as a live-attenuated vaccine against acute, chronic, and congenital *Toxoplasma gondii* infection in mice. Immune response evoked by Δ*GRA17* immunization suggested a sequential Th1 and Th2 T cell response, indicated by high levels of Th1 and a mixed Th1/Th2 cytokines at 28 and 70 days after immunization, respectively. Δ*GRA17*-mediated immunity fully protected mice against lethal infection with wild-type (wt) RH strain, heterologous challenge with PYS, and TgC7 strains of the Chinese ToxoDB#9 genotype, and *T. gondii* Pru strain. Although parasite cysts were detected in 8 out of 10 immunized mice, cyst burden in the brain was significantly reduced (*P* < 0.05) in immunized mice (53 ± 15 cysts/brain) compared to non-immunized mice (4,296 ± 687 cysts/brain). In respect to congenital infection, the litter size, survival rate, and body weight (BW) of pups born to Δ*GRA17*-immunized dams were not different compared to pups born to naïve control dams (*P* = 0.24). However, a marked reduction in the litter size (*P* < 0.001), survival rate, and BW (*P* < 0.01) of pups born to non-immunized and infected dams was detected. Also, immunized dams infected with type II Pru strain had significantly (*P* < 0.001) less cyst burden in the brain compared with non-immunized and infected dams. These findings show that immunization with Δ*GRA17* strain evokes cell-mediated and neutralizing antibody responses and confers some degree of protection against challenge with homologous and heterologous virulent *T. gondii* strains.

## Introduction

The obligate intracellular apicomplexan protozoan *Toxoplasma gondii* is capable of infecting almost virtually all warm-blooded animals and has been estimated to chronically infect one-third of the world’s population (1). Infection of humans and animals (intermediate host) occurs by ingestion of either tissue cysts (containing bradyzoites) in undercooked meat or oocysts (containing sporozoites) that are shed in the feces of cat (definitive host). The pathogenesis of *T. gondii* infection consists of a primary infection at the site of exposure, transport of the parasite into many body organs especially the nervous system where acute and latent infection develop. *T. gondii* infection is often asymptomatic in immunocompetent individuals; however, the parasite can cause serious health consequences in immunocompromised individuals, such as AIDS patients (2–4). Primary or reactivated toxoplasmosis during pregnancy put fetuses of infected dams at risk of congenital infection, with manifestations ranging from retinitis to hydrocephalus and cognitive impairment (3, 5, 6). However, infection with *T. gondii* before pregnancy may elicit protective immunity against subsequent parasite challenge, underscoring the benefit of vaccination prior to pregnancy to stimulate immune response that protects against congenital transmission (6–8).

The worldwide distribution of *T. gondii* (1), the lack of human vaccine, the side-effects of current therapeutics (9, 10), and their inability to eliminate the tissue cysts, and the emergence of anti-*T. gondii* resistant strains (11) necessitate the development of new interventions to effectively control and prevent toxoplasmosis (12–14). Several vaccine strategies (e.g., inactivated, subunit, and DNA vaccines) against *T. gondii* have been described, but none of them was able to provide full protection. The use of live-attenuated strain is particularly promising because it can induce more protective cellular and humoral immunity, simulating natural infection without causing the disease (12–14). Toxovax^®^, the only available commercial vaccine, is based on live-attenuated *T. gondii* S48 strain and is licensed only for use in sheep to prevent abortion (15).

A few attempts have been made to generate attenuated *T. gondii* strains unable to cause disease *via* removal of virulence or metabolic factors using targeted gene deletion (16). For instance, mitogen-activated protein kinase 1 has been shown to be critical for bradyzoite differentiation, attachment, and replication *in vitro*, and virulence in mice (17). Also, some dense-granule protein (GRA2, GRA3, and GRA6) mutants have been shown to exhibit reduced virulence (18–20). The non-replicating and non-cyst-forming uracil auxotroph Δ*ompdc* mutants were shown to induce protective immunity against acute and chronic *T. gondii* infection in mice (21). Mic1-3KO strains lacking both *mic1* and *mic3* genes were able to induce protective immunity against chronic and congenital toxoplasmosis in mice, and against *T. gondii*-induced abortion in sheep (22, 23). Dense granule protein 17 (*GRA17*) gene has been shown to maintain the structural stability of the parasitophorous vacuole (PV) and mediates the transport of molecules across the PV membrane (24). Targeted deletion of Δ*GRA17* reduced the ability of mutant strains to proliferate *in vitro* or to cause disease in mice (24), indicating that *GRA17* gene is an essential virulence factor with a potential immunogenicity.

In the vaccine studies presented here, we investigated the immunogenicity and protective efficacy of *T. gondii* RH Δ*GRA17* mutant strain, in the Kunming mouse model and characterized the protecting humoral and cellular immune responses. We examined the immune responses protective against not only the lethal infection with wild-type (wt) RH strain but also against heterologous local strains (PYS and TgC7) of the Chinese ToxoDB#9 genotype. Furthermore, the efficacy of immunization with Δ*GRA17* mutant strain was tested against acute, latent, and congenital infections. Interpretation of the data and potential immune mechanisms involved in protection are discussed.

## Materials and Methods

### Ethics Statement

All animal protocols were reviewed and approved by the Animal Administration and Ethics Committee of Lanzhou Veterinary Research Institute, Chinese Academy of Agricultural Sciences. The study was performed in strict compliance with the recommendations set forth in the Animal Ethics Procedures and Guidelines of the People’s Republic of China. All efforts were made to minimize animal suffering and to reduce the numbers of animals used in the experiments.

### Mice

*Toxoplasma gondii*-seronegative 8-week-old female and male Kunming mice were purchased from Lanzhou University Laboratory Animal Center, Lanzhou, China. They were housed under pathogen-free conditions at Lanzhou Veterinary Research Institute in controlled room under stable conditions (12-h/12-h dark/light cycle, 50–60% humidity and 22°C temperature). Mice had free access to sterilized food and water *ad libitum*. Mice were acclimated for 1 week before use. Kunming mice were selected because of their susceptibility to acute and latent infection with *T. gondii* (14). In all experiments, mice (6–10 mice per group) were observed daily after immunization and challenge.

### Preparation of Different Parasite Stages

Tachyzoites of *T. gondii* type I RH mutant and wt strains and type ToxoDB#9 − PYS and TgC7 − strains were maintained *in vitro* in human foreskin fibroblast (HFF, ATCC SCRC-1041) monolayers as previously described (25). Freshly egressed tachyzoites were harvested from HFF monolayers by filtration through 3-µm polycarbonate membranes and suspended in sterile phosphate-buffered saline (PBS). *T. gondii* cysts of type II Prugniuad (Pru) strain were maintained in Kunming mice by oral passage of infectious cysts in mice as described previously (26). The number of parasites (tachyzoites or cysts) of each strain was adjusted in PBS to the final number used in the experiments.

### Preparation of Soluble *Toxoplasma* Antigens (STAg)

Soluble *Toxoplasma* antigens were prepared as previously described (27). Briefly, a suspension of *T. gondii* RH tachyzoites was washed in PBS and subjected to repeated freezing and thawing cycles and then sonicated on ice at 60 W/s. The preparations were centrifuged at 14,000 g for 30 min at 4°C. The supernatants were sterile filtered with 0.22 µm sterile nitrocellulose filters (Sartorius) and the STAg concentration was determined by the Bicinchoninic acid kit (BCA, Sigma, St. Louis, MO, USA), and aliquoted was stored at −80°C until use.

### Generation of Δ*GRA17* Strain Using CRISPR-Cas9

Disruption of *GRA17* gene in RH strain has been shown to affect the parasite’s ability to proliferate in cultured cells and its virulence in mice (24). We used CRISPR-Cas9 method to delete *GRA17* gene in *T. gondii* RH strain as described previously (28, 29). Briefly, we generated a single-guide RNA (sgRNA) to disrupt *GRA17* gene and modified pSAG1:CAS9-U6:sgUPRT by PCR mutagenesis using specific primers (see Table S1 in Supplementary Material). The DHFR* resistance cassette was amplified as described previously (28, 29). Then, *GRA17*-specific CRISPR-Cas9 plasmid and the DHFR* resistance cassette amplicons were combined and transfected into *T. gondii* RH tachyzoites. After selection with 3 µM pyrimethamine, single clones were screened by PCR using specific primers (KO-GRA17-F and KO-GRA17-R) listed in Table S1 in Supplementary Material. Then, we compared the *in vitro* growth of Δ*GRA17* deletion mutant to wt *T. gondii* tachyzoites. Briefly, confluent monolayers of HFF grown on glass coverslips positioned in the bottom of 6-well plates were infected with freshly purified tachyzoites (2 × 10^3^/well) of mutant or wild-type (wt) strain. Coverslips were washed to remove extracellular parasites at 3 h after infection, and the parasite’s growth within the PV was assessed at 12 and 24 h by fixation in 4% paraformaldehyde followed by imaging at ×40 using a Leica DM IRB microscope equipped with a high-resolution charge-coupled device camera (Nikon Japan).

### Immunization of Mice

Mice were immunized with 5 × 10^4^ Δ*GRA17* tachyzoites or mock-immunized in a volume of 200 µl PBS intraperitoneally (i.p.) using a 26-gauge needle (Figure S1 in Supplementary Material). We used i.p. immunization because it can induce both mucosal and systemic immune responses. The type I RH strain was used because immunization with Δ*GRA17* RH tachyzoites has been shown to protect mice from lethal infection (24).

### Immune Responses in Pre-challenged Mice

We assessed the immune response in Δ*GRA17*-immunized mice prior to infection in order to determine the pattern of humoral and cellular immune responses of the vaccinated mice compared to non-immune control animals.

#### Detection of Total IgG and Antibody Isotypes in Δ*GRA17*-Immunized Mice

Blood samples were collected from the mouse tail vein at 28 and 70 days after immunization. Serum samples were collected after clot retraction and were stored at –80°C until analysis. The total and subclasses of anti-*T. gondii* IgG antibodies as an indicator of Th1 and Th2 responses were determined by ELISA as previously described (30, 31). Briefly, wells of 96-well microtiter plates were coated with 100 µl (10 µg/ml) STAg (diluted in PBS) and were incubated at 4°C overnight. Each well was washed with PBS-Tween 0.05% (PBS-T) and blocked with PBS-T plus 1% low fat milk for 1 h at ambient temperature. Then, the plates were washed three times in PBS. Then, 100 µl serum samples were added to the wells at a dilution of 1:25 with PBS and were incubated for 1 h at 37°C. The plates were washed three times with PBS, followed by addition of 100 µl of horseradish-peroxidase-conjugated goat anti-mouse IgG (1:250 diluted with PBS), anti-mouse IgG1 (1:500 diluted with PBS), and IgG2a (1:500 diluted with PBS). The plate was further incubated for 1 h at 37°C. After washing three times, 200 µl substrate solution (1.05% citrate substrate buffer, 0.03% H_2_O_2_, 1.5% ABTS) was added for 20 min and the absorbance at 450 nm was read using ELISA plate reader (iMark microplate absorbance reader; Bio-Rad, Hercules, CA, USA).

#### Cytokine Analysis in Splenocyte Supernatants

Seventy days after immunization, mice were sacrificed and spleens were removed aseptically into RPMI medium in a class II hood and single-cell suspensions were prepared. Briefly, the spleen was teased gently through a tea strainer in a petri dish in complete RPMI medium. The resultant suspensions including splenocytes were harvested with a sterile Pasteur pipette allowing tissue debris to sediment. All separated spleen cell suspensions were centrifuged for 10 min at 1,200 *g* at 4°C, and resuspended in 0.3 ml of Boyle’s solution (one volume of 0.17 M tris–HCl pH 7.65 and nine volumes of 0.16 M ammonium chloride) to lyse erythrocytes. After 3 min incubation, at ambient temperature, the spleen cells were centrifuged at 1,200 *g* for 5 min at 4°C, washed three times with RPMI, and the pellets were resuspended in 10 ml of complete RPMI medium. The number of viable splenocytes was calculated using Trypan blue exclusion assay and Neubauer Haematocytometer under phase contrast microscope. Dead cells did not exclude the dye. One million splenic cells collected from mice in different groups were seeded in triplicate wells in sterile 96-well flat-bottom tissue culture plates (Corning Incorporated, Corning, NY, USA) in a final volume of 200 µl RPMI supplemented with 10% heat-inactivated fetal calf serum, 2 mM glutamine, and 1% penicillin–streptomycin mixture. STAg or medium alone was added to the culture at a final concentration of 10 µg/ml. Culture supernatants were collected and the level of the secreted interleukin-2 (IL-2), IL-12, IL-10, and interferon gamma (IFN-γ) was determined using a commercial ELISA (eBioscience^®^ Bender MedSystems GmbH, Austria).

### Challenge Models to Test the Efficacy of Immunization

In these experiments, the efficacy of immunization with Δ*GRA17* mutant strain was tested against acute, latent, and congenital infections in mice (Figure S1 in Supplementary Material).

#### Protection against Acute Infection

In this experiment, we investigated the immune-protection of Δ*GRA17* tachyzoites against acute *T. gondii* infection. Briefly, 70 days after immunization, mice were challenged with 1 × 10^3^ RH tachyzoites of *T. gondii* RH, ToxoDB#9 PYS, or ToxoDB#9 TgC7 strain. ToxoDB#9 genotype is the predominant genotype in China and has a similar virulence to Type I RH (32). Therefore, it was important to determine if immune responses elicited against the Δ*GRA17* mutant protects against challenge with the heterologous local strains (PYS and TgC7) of ToxoDB#9 genotype. Seven days postinfection (dpi), serum and peritoneal fluid were collected from Δ*GRA17*-immunized and RH-infected mice, non-immunized and RH-infected mice, and non-immunized and uninfected mice. Then, the level of pro-inflammatory cytokines IFN-γ and IL-12 in the the collected sera and peritoneal washes was assessed by ELISA following the manufacturer’s instructions (eBioscience^®^ Bender MedSystems GmbH, Austria). Pre-immunization blood samples served as a control.

#### Protection against Chronic Infection

We also evaluated the level of protection induced by Δ*GRA17* mutant against chronic infection in mice challenged with 20 cysts of *T. gondii* Pru strain. Chronically infected and immunized mice and control mice were euthanized at 35 dpi and their brains were removed and individually homogenized in 1 ml of PBS. The parasite cyst burden in the brain tissues of the mice was performed by examining dilutions of DBL-stained brain homogenates using a Zeiss wide-field epifluorescence microscope with 10× objective as previously described (33). Brain homogenates that did not contain cysts upon microscopic examination were further bio-assayed in mice. Briefly, PBS-homogenized brain tissues were used to infect orally healthy mice (one brain homogenate per mouse). Five weeks later, blood samples were collected for detection of anti-*T. gondii* antibodies using ELISA as described above. Survival of acutely or chonically infected and immunized mice versus non-immunized and infected mice was evaluated using Mantel–Cox log-rank test.

#### Protection against Congenital Toxoplasmosis

Seventy days post immunization female mice were mated to males, at two females to one male ratio, and examined every 12 h after copulation for evidence of a vaginal plug. The date on which a plug was observed was considered day 1 of pregnancy, at which time pregnant females were separted from male mice. Then, we compared between five groups of pregnant mice: (group A) Δ*GRA17*-immunized and orally gavaged with 10 tissue cysts of *T. gondii* Pru strain on day 12 of gestation, (group B) Δ*GRA17*-immunized and i.p. inoculated with 200 *T. gondii* RH tachyzoites on day 18 of gestation, (group C) non-immunized, and orally gavaged with 10 tissue cysts of *T. gondii* Pru strain on day 12 of gestation, (group D) non-immunized and i.p. inoculated with 200 *T. gondii* RH tachyzoites on day 18 of gestation, and (group E) non-immunized and uninfected. The level of protection against congenital infection was determined by analyzing litter size and survival rate of the naturally delivered pups at birth and 5 days post-delivery, and body weight (BW) at 5 days of age in i.p. RH-infected mice, and at birth and 35-day old in orally Pru-infected mice. Also, the level of protection in Pru-infected mice was evaluated by quantifying the parasite cyst burden in the brain tissue in the survived pups at 35 days of age and in their dams at 30 days after delivery. Maternal splenocytes were obtained from pregnant mice infected with Pru cysts on day 12 of gestation and were stimulated with STAg 6 days later (i.e., 18 days of gestation). Then, the level of IFN-γ, IL-2, IL-12 (Th1), and IL-10 (Th2) cytokines in immunized and infected mice, non-immunized and infected mice, and non-immunized and uninfected mice was determined as described above.

### Statistical Analysis

All statistical analyses were performed using SPSS18.0 (SPSS Inc., Chicago, IL, USA). Levels of significance of the differences in the level of antibodies, cytokines, and parasite cyst burdens were compared using one-way ANOVA analysis (for comparing means between ≥three groups) or two-tailed, unpaired Student’s *t-*test (for comparing means between two groups). The SDs were derived from three independently performed experiments with three replicates per experiment for the *in vitro* assays. Statistical significance was set at *P* < 0.05. Mortality was examined by plotting survival curves of the different mouse groups stratified by *T. gondii* infection and immunization status using Mantel–Cox log-rank test.

## Results

### Generation of *GRA17*-Deficient Type I *T. gondii*

**Figure 1:**
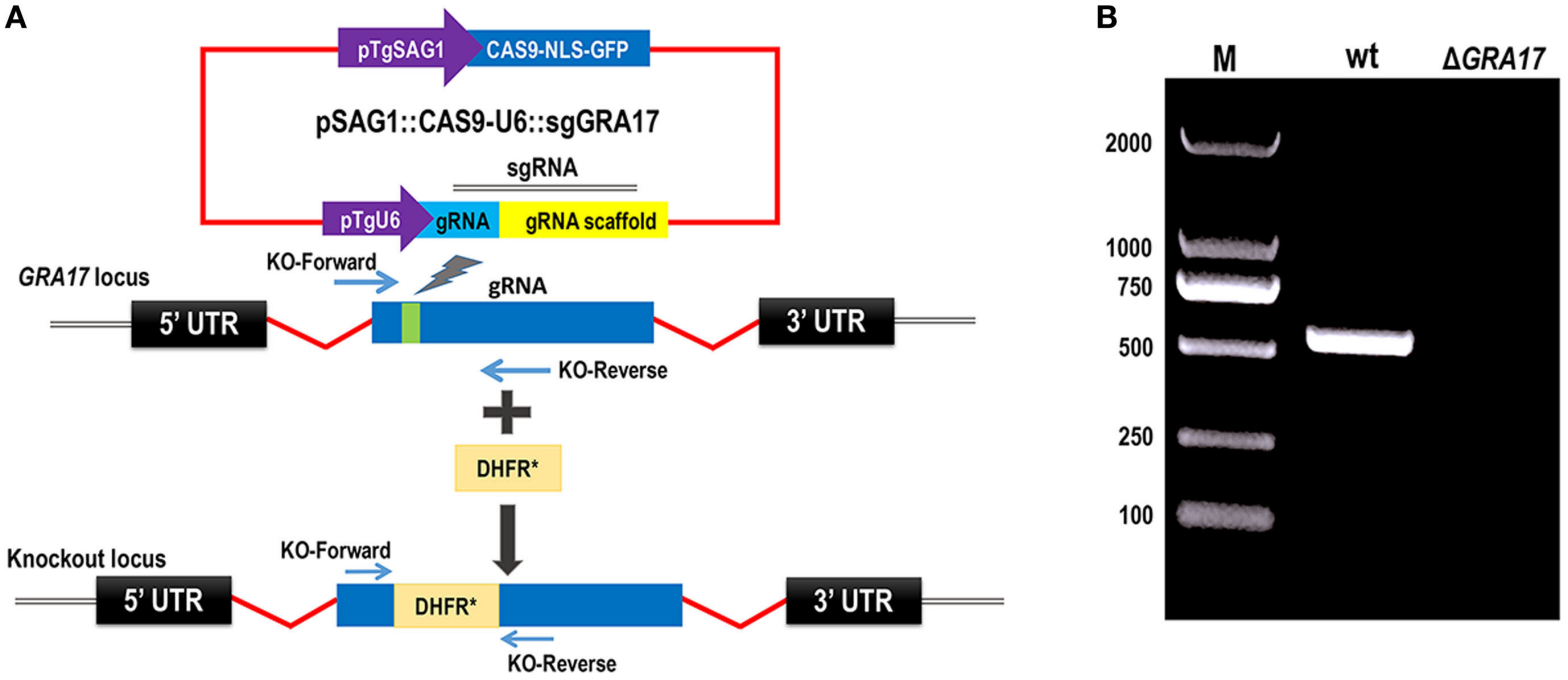
CRISPR/Cas9-mediated disruption of *GRA17* gene in *Toxoplasma gondii* Type I RH strain. **(A)** Schematic representation of CRISPR-Cas9-mediated disruption of *GRA17* gene by insertion of pyrimethamine-resistant DHFR* cassette into *GRA17*. Transfection of pSAG1:CAS9-U6:sgGRA17 together with the DHFR* amplicon was used to disrupt the *GRA17* coding region. **(B)** KO forward and KO reverse primers were used to amplify the small fragment. Diagnostic PCR showing DHFR* cassette integration in a representative *GRA17*:DHFR* clone compared with the parental wild-type RH strain (wt). M indicates the molecular weight marker.

**Figure 2:**
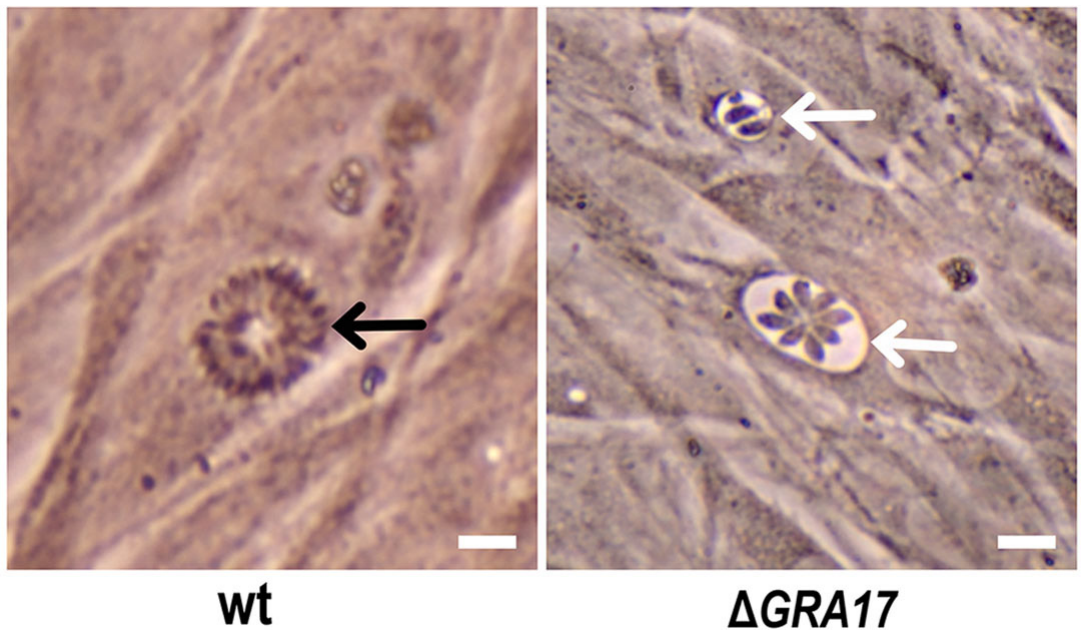
Phenotypic analysis of Δ*GRA17*-infected human foreskin fibroblast cells. Δ*GRA17* mutant developed deformed parasitophorous vacuoles with limited parasite proliferation capability (white arrows) compared to the normal intra-vacuolar tachyzoite’s growth and vacuole’s morphology exhibited by the wt strain (black arrow), indicating the successful deletion of the *GRA17* gene. Scale bars, 10 µm.

**Figure 3:**
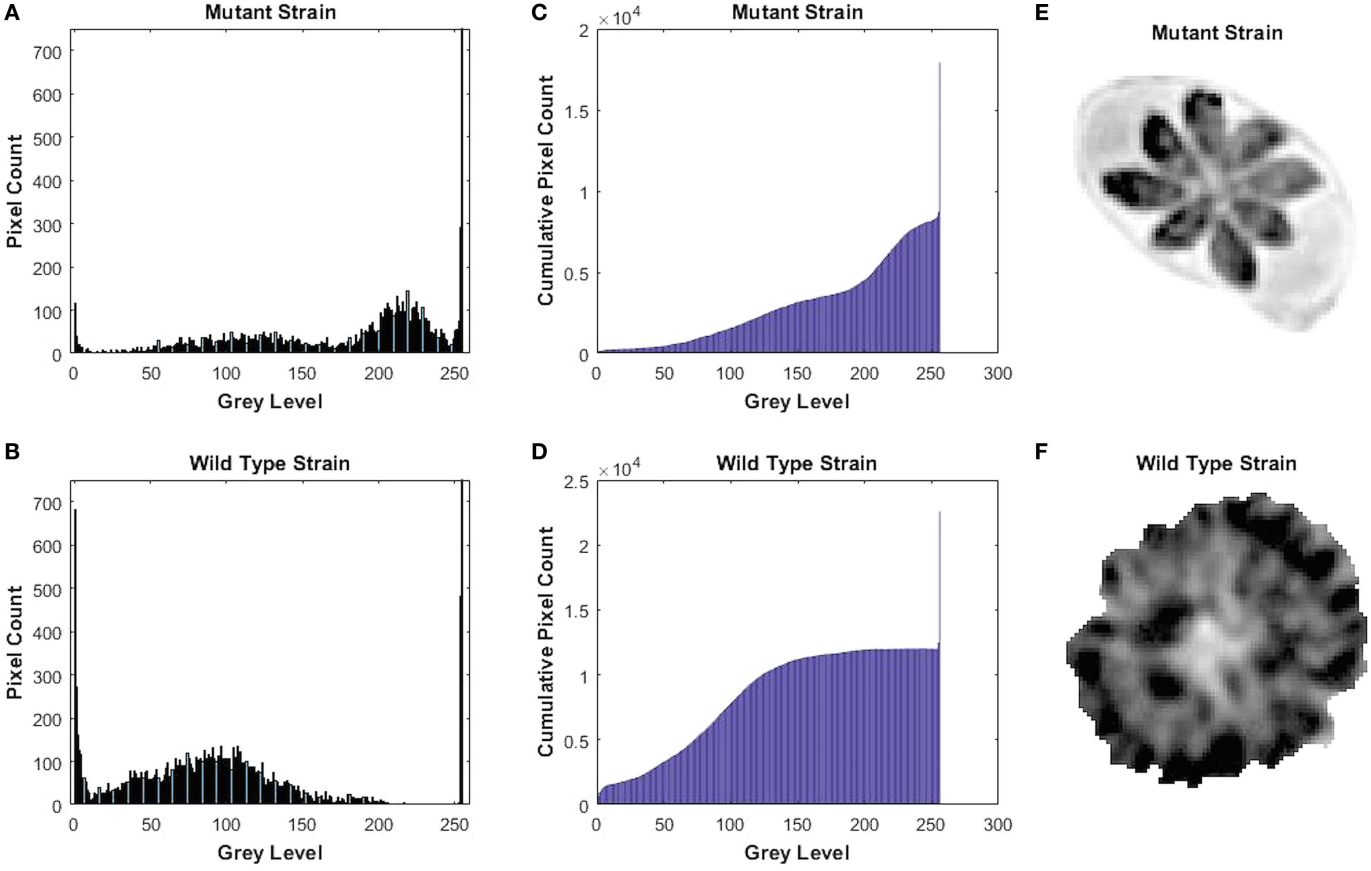
Gray-level histogram for mutant and wild-type *Toxoplasma gondii* strains. **(A,B)** show the number of pixels in each gray level (0–256) of the images of the parasitophorous vacuole (PV) with 0 signifying black and 256 signifying white. **(C,D)** show the cumulative pixel count for the same images, which are shown in **(E,F)**. This shows that PV produced by wild-type strain had a significantly larger fraction of darker pixels, which represents the large number of the growing intra-vacuolar parasites, whereas the PV of mutant strain had significantly lower quantity of the intra-vacuolar parasites, as shown by a greater proportion of the ligher pixels.

We knocked out *GRA17* gene to study whether *GRA17*-deficient RH tachyzoites can be used as a live-attenuated vaccine against acute, chronic, and congenital toxoplasmosis in mice. CRISPR-Cas9 system was used to delete the *GRA17* coding sequence (Figure 1A). The sgRNA and DHFR* marker were inserted into the *GRA17* coding sequence and stable, single clones were successfully generated and validated by PCR (Figure 1B). We observed a significant defect in the appearance of the PV, along with less intra-vacuolar tachyzoite’s proliferation in the Δ*GRA17* mutant compared to the wt strain (Figure 2). Gray-level histogram analysis showed that the PV produced by wild *T. gondii* RH strain had a significantly larger fraction of darker pixels, which represent the large number of growing intra-vacuolar parasites, whereas a significantly lower number of parasites was observed in the PV produced by the Δ*GRA17* mutant strain, as shown by a greater proportion of the lighter pixels (Figure 3).

### Immune Responses of Mice to Immunization

**Figure 4:**
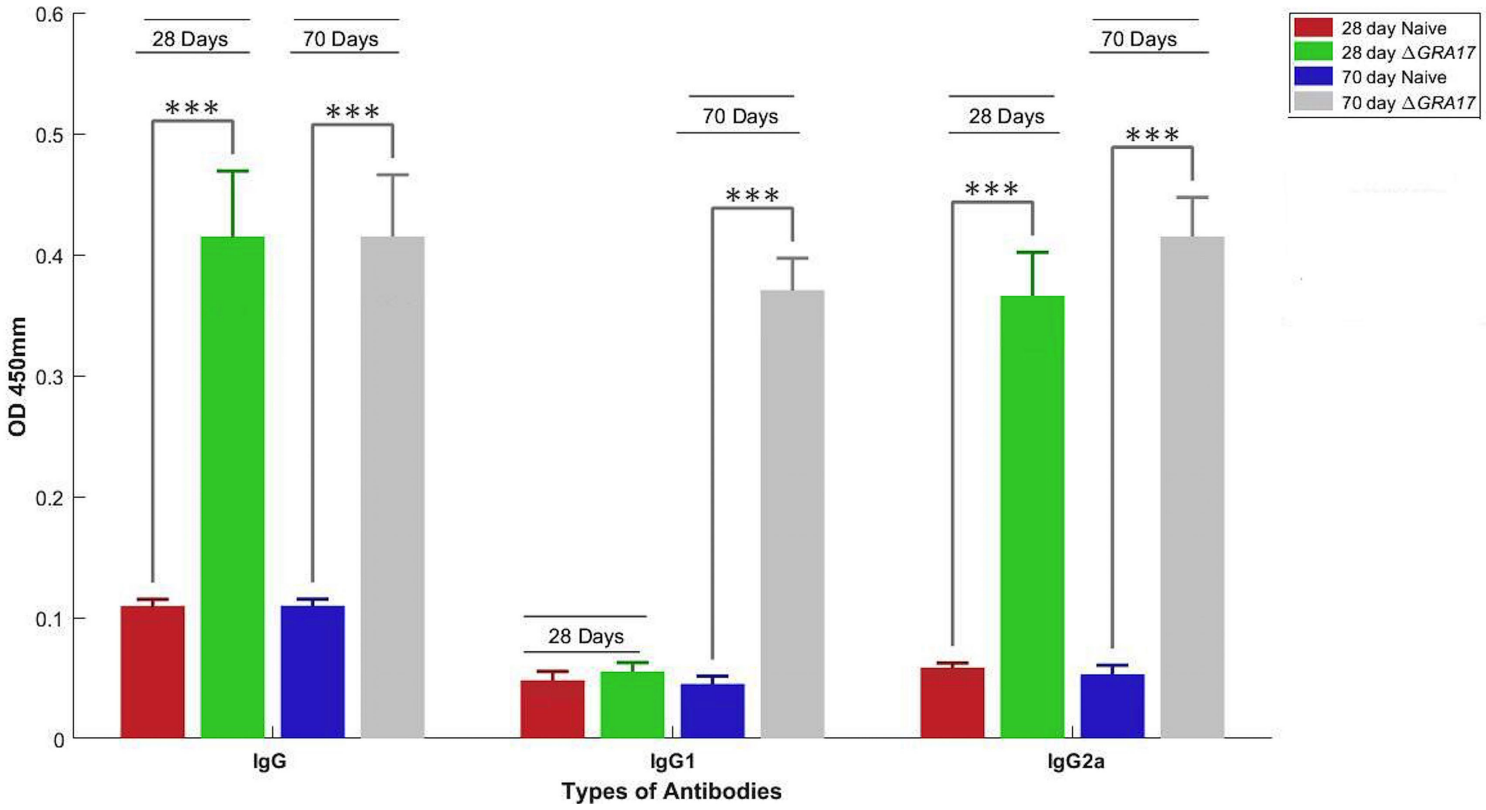
Humoral response and antibody isotype profile in serum of mice immunized with Δ*GRA17* by intraperitoneal route. Levels of IgG and IgG subclass (IgG1 and IgG2a) antibodies in the sera of mice at 28 and 70 days after Δ*GRA17*-immunization compared to naïve mice. The patterns of IgG2a to IgG1 at 28 and 70 days postimmunization suggest the induction of a Th1 immune response at 28 days postimmunization, which remained high and the induction of Th2 immune response at day 70 postimmunization. Results are expressed as mean of OD450 ± SD. Significance compared with naïve control mice: ***p < 0.001.

**Figure 5:**
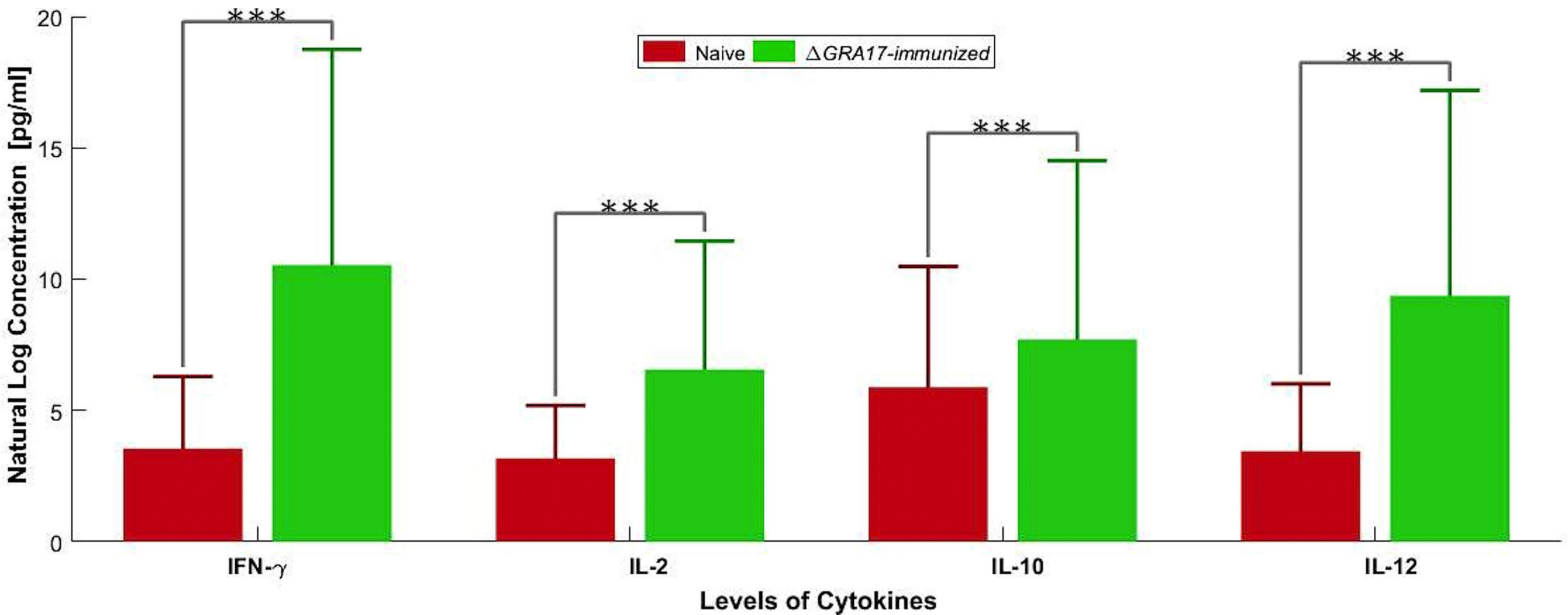
Levels of Th1 [IFN-γ, interleukin-2 (IL-2), and IL-12] and Th2 (IL-10) cytokines produced by spleen cells of Δ*GRA17*-immunized mice. Spleen cells from mice were obtained 70 days postimmunization and were stimulated *in vitro* with soluble *Toxoplasma gondii* tachyzoite antigen (10 µg/ml). Cell-free supernatants were harvested and evaluated for IL-2, IL-10, IL-12, and INF-γ using ELISA. Significance compared with naïve control mice: ***p < 0.001.

The prechallenge sera from the immunized mice were monitored for their IgG subclasses and for Th1 and Th2 cytokine profiles. The presence of specific anti-*T. gondii* IgG antibodies at 28 and 70 days after immunization was determined by quantitative ELISA. At day 28 postimmunization, all immunized mice had seroconverted with a higher level of IgG antibodies compared with naïve mice (Figure 4). Next, we tested whether a Th1 and/or Th2 response was elicited in the immunized mice by evaluating the levels of specific antibodies, IgG1 and IgG2a. At 28 days postimmunization, the levels of IgG2a in immunized mice were significantly higher compared to control naïve mice. In contrast, levels of IgG1 did not increase in immunized mice compared with naïve mice (Figure 4). At 70 days postimmunization, the levels of IgG1 in immunized mice increased, and the levels of IgG and IgG2a remained higher compared to naïve mice (Figure 4). Cytokines released from spleen cells after stimulation with STAg were evaluated by ELISA. As shown in Figure 5, the levels of Th1-type cytokines (IFN-γ, IL-2, and IL-12) in Δ*GRA17*-immunized mice were significantly higher than that in naïve mice. In the interim, the level of Th2-type cytokine (IL-10) was significantly increased in supernatants from spleen cells of Δ*GRA17*-immunized mice compared to naïve mice.

### Protection against Acute Infection

**Figure 6:**
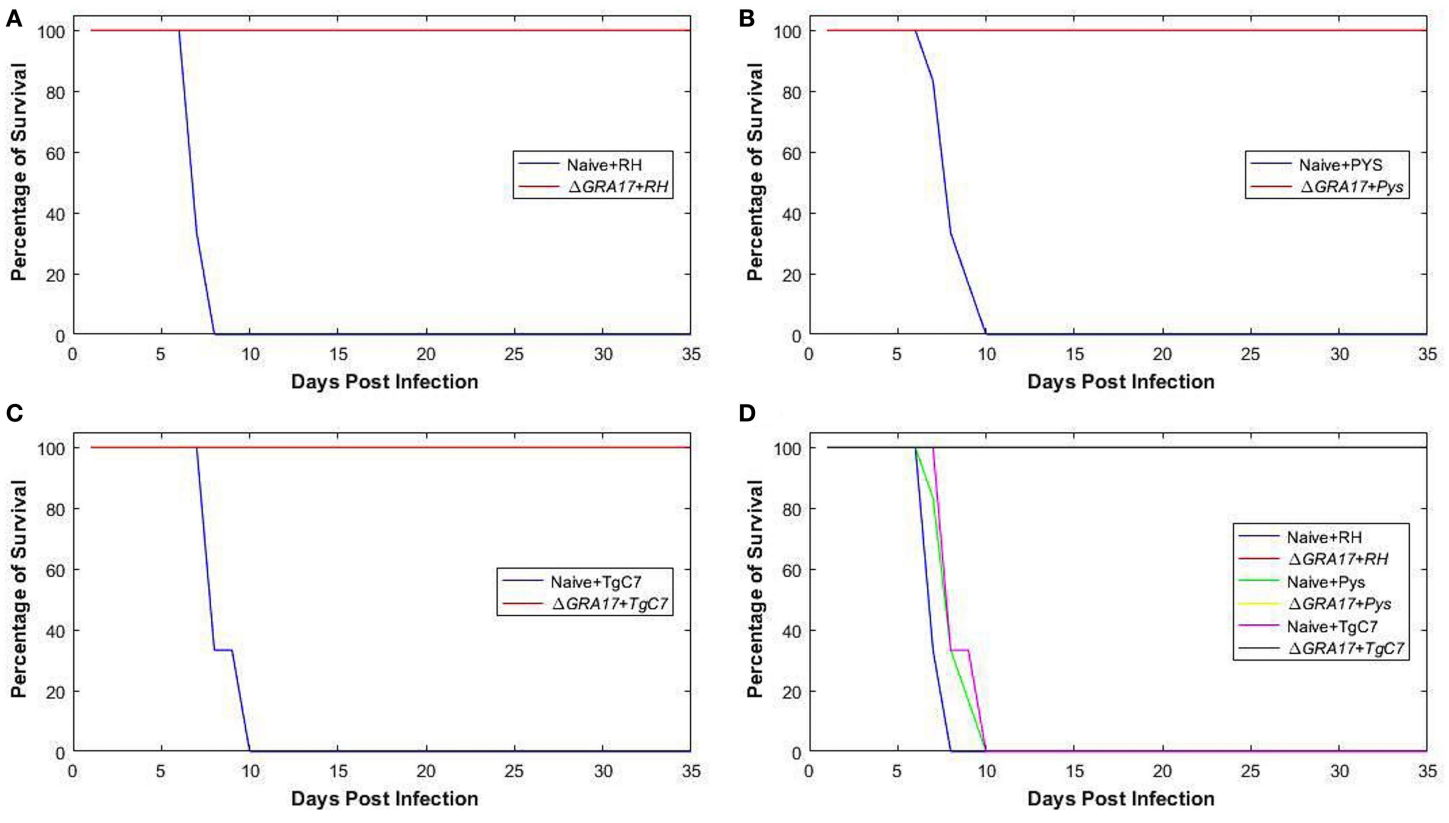
Protection of mice against acute lethal *Toxoplasma gondii* infection. Survival curves of Δ*GRA17*-immunized mice, challenged intraperitoneally with 1 × 10^3^ tachyzoites of RH, PYS, or TgC7 strain 70 days after immunization. The survival of mice was monitored for 35 days. A Log-rank (Mantel–Cox) test revealed that the difference in the survival rates between immunized and infected groups compared to non-immunized and infected groups as *P* = 0.003. All mice in the immunized groups remained alive at day 35 after infection, but all mice in the infected and non-immunized groups died between day 7 and 10 after infection. In addition to separate curves that show the difference in the survival rate between immunized and non-immunized mouse groups infected with RH, PYS, or TgC7 strain **(A–C)**, the overall survival rates in all immunized groups compared with the non-immunized positive control groups were combined in one graph **(D)**.

**Figure 7:**
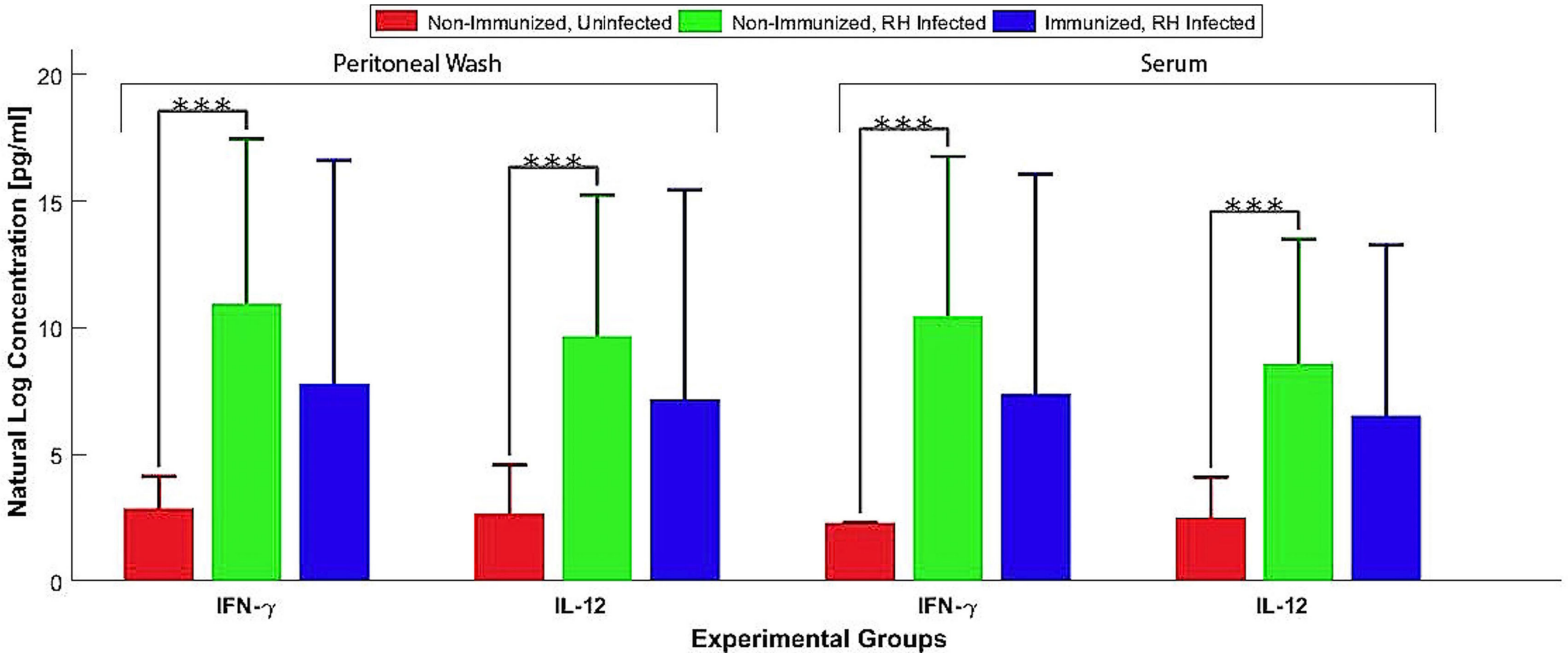
Pro-inflammatory cytokines produced by Δ*GRA17-*immunized mice after challenge with *Toxoplasma gondii* RH. Mice were challenged with 1 × 10^3^ RH tachyzoites 70 days postimmunization and the levels of IFN-γ and IL-12 in the serum and peritoneal washes were assessed by ELISA at 7 days postinfection. Highest levels of pro-inflammatory cytokines were found in the non-immunized and infected mice. Significance compared with naïve control mice: ***p < 0.001.

Kunming mice, 70 days post Δ*GRA17* immunization, were challenged with 1 × 10^3^ tachyzoites of *T. gondii* RH, PYS, or TgC7 strain. All immunized mice survived the challenge with the three strains. By contrast, all naïve mice challenged with RH, PYS, or TgC7 strain died within 10 dpi (Figure 6). Assessment of cytokine response after chgallenge with with RH was performed 7 dpi using ELISA. Analysis was performed on serum and peritoneal fluids from Δ*GRA17*-immunized and infected mice, non-immunized and infected mice, and non-immunized and uninfected mice. The levels of IFN-γ and IL-12 in the sera and peritoneal washes (Figure 7) were significantly higher in non-immunized and infected mice than in the immunized and infected, and non-immunized and uninfected mice.

### Protection against Chronic Infection

**Figure 8:**
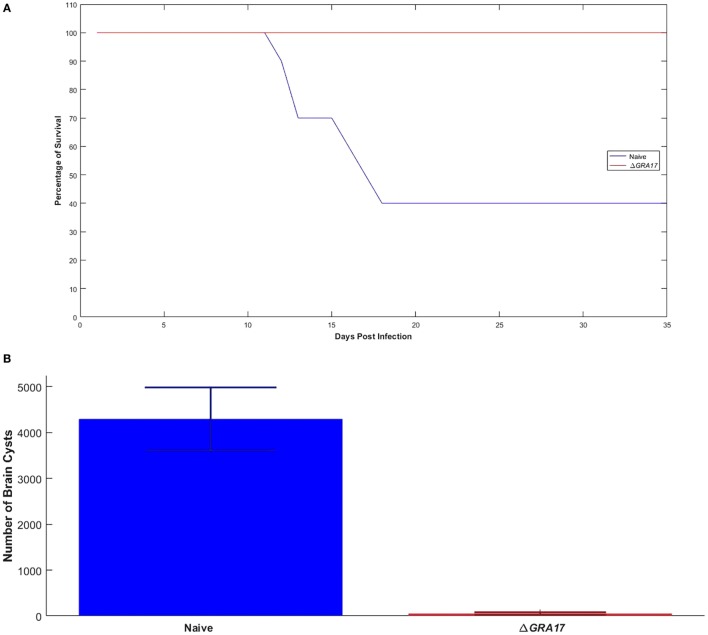
Immunization with Δ*GRA17* promotes survival and inhibits brain cyst burden after infection. **(A)** Survival curves following challenge of Δ*GRA17*-immunized mice with 20 cysts of *Toxoplasma gondii* type II Pru strain, compared with mock-immunized mice, 70 days after immunization. The survival of mice was monitored for 35 days. A Log-rank (Mantel–Cox) test demonstrated a significant difference between the immunized and control groups (*P* = 0.0001). **(B)** Cyst burden in the brain of mice that survived to 35 days after challenge (Δ*GRA17*-immunized mice vs infected, non-immunized mice). Data points indicate means ± SD.

Seventy days after immunization, both immunized and control mice were orally gavaged with 20 cysts of *T. gondii* Pru strain. All Δ*GRA17*-immunized and Pru-infected mice survived compared to only 40% survival of non-immunized and infected mice (Figure 8A). Parasite cyst burden in the brains 5 weeks post-challenge with *T. gondii* Pru cysts was determined. Infected mice had a larger number of cysts (4,296 ± 687 cysts/brain) compared with immunized mice that had significantly less cyst burden (53 ± 15 cysts/brain) (*P* < 0.05). Brain cysts were not found in 4 out of 10 (40%) immunized mice by microscopic examination (Figure 8B). Brain tissues of these four immunized mice that did not show any cysts were homogenized and bio-assayed in naïve mice. Anti-*T. gondii* IgG antibodies were found in two of these four naïve mice suggesting that two brain homogenates derived from immunized and infected mice are infected based on serological evidence and two were completely free of parasites as evidence by negative microscopic and serological results.

### Protection against Congenital Toxoplasmosis

Immunization with Δ*GRA17* was well tolerated during pregnancy as evidenced by the absence of abortion in immunized mice, number of pups per litter, survival rate, and BW of pups born to immunized dams. Also, it is important to note that an immunopathologic response was not induced by immunization with Δ*GRA17* strain.

#### Protection against Type I RH Strain Infection during Pregnancy

**Figure 9:**
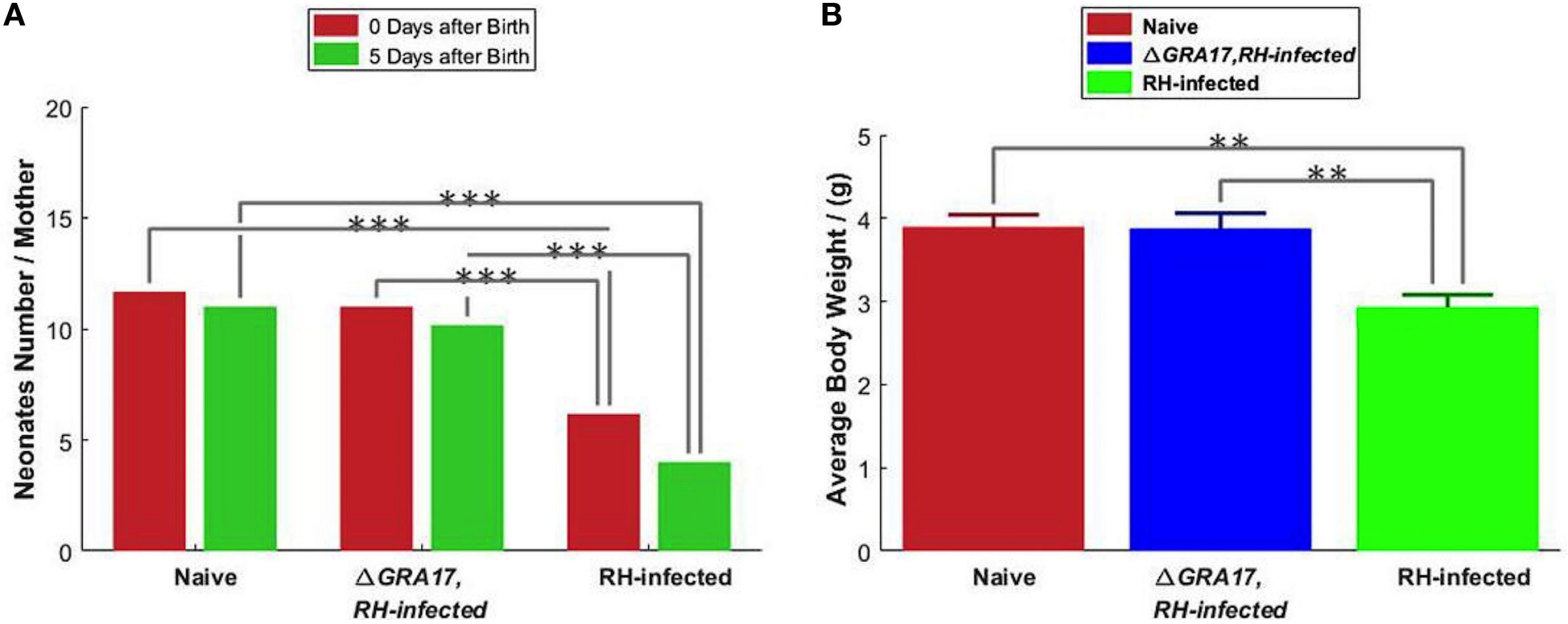
Protection of mice against type I RH tachyzoite infection on day 18 of gestation. **(A)** The litter size of pups from the non-immunized and uninfected mice (naïve); non-immunized and RH-infected mice, and immunized and RH-infected mice was assessed at birth and 5 days after birth. **(B)** The body weight of 5-day-old pups. Significance compared with naïve control mice: **p < 0.01, ***p < 0.001.

Female mice were mated with males on day 70 after immunization and pregnant mice were infected with 200 RH tachyzoites on day 18 of gestation, 3 days before neonates were born. Neonates were counted at birth and visually inspected for appearance and sizes. The mean sizes of viable neonates born to immunized and infected mice were similar to pups born to non-immunized and uninfected mice and in both groups, no abortions were observed. In contrast, the mean litter sizes of viable neonates born to non-immunized and infected mice were significantly reduced compared to pups from immunized and infected mice, and abortions were also observed (Figure 9A). Five days after birth, the survival rate and mean BW of pups born to non-immunized and uninfected, and immunized and infected mice were significantly higher than that from non-immunized and infected mice (Figure 9B). Non-immunized and infected dams showed clinical signs of toxoplasmosis, such as weight loss, weakness, anorexia, hunched posture, and ruffled fur, which became profound by day 7 after challenge. In contrast, immunized and infected, and non-immunized and uninfected dams did not show any clinical signs (i.e., exhibited normal physical conditions). Some non-immunized and infected dams that were unable to eat and drink were sacrificed (and their pups) on day 5 after birth to detect the parasites DNA in their spleen and livers. *T. gondii* DNA was found in all non-immunized and infected dams, but not in any of the 10 examined pups. However, parasites DNA was detected in some aborted fetuses. The pups from the immunized and infected mice had equivalent mean BW and survival rate to that from non-immunized and uninfected dams on day 35 of age (date not shown).

#### Protection against Type 2 Pru Strain Infection during Pregnancy

**Figure 10:**
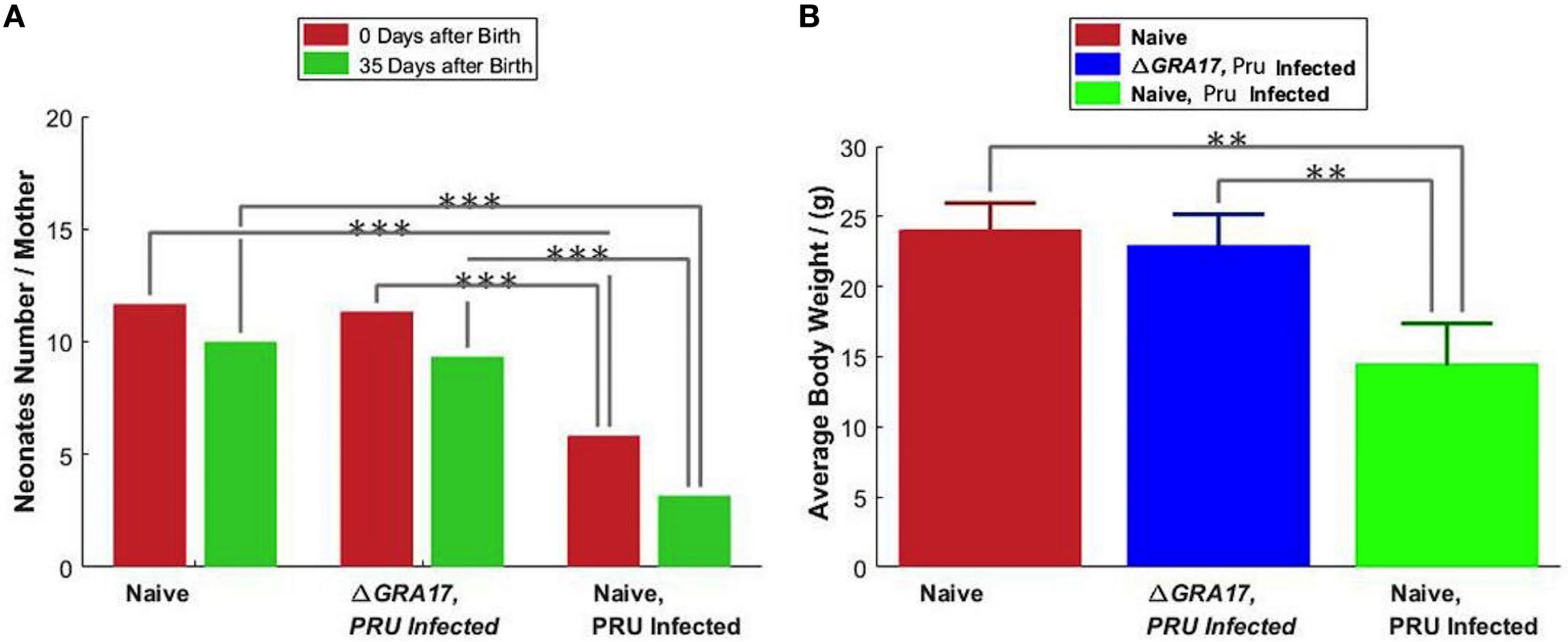
Protection of mice against type II Pru cysts infection on day 12 of gestation. **(A)** The litter size of pups from the non-immunized and uninfected mice (naïve), non-immunized and Pru-infected mice, and immunized and Pru-infected mice was assessed at birth and 35 days after birth. **(B)** The body weighs of 35 days old pups. Data points indicate means ± SD. Significance compared with naïve control mice: **p < 0.01, ***p < 0.001.

**Figure 11:**
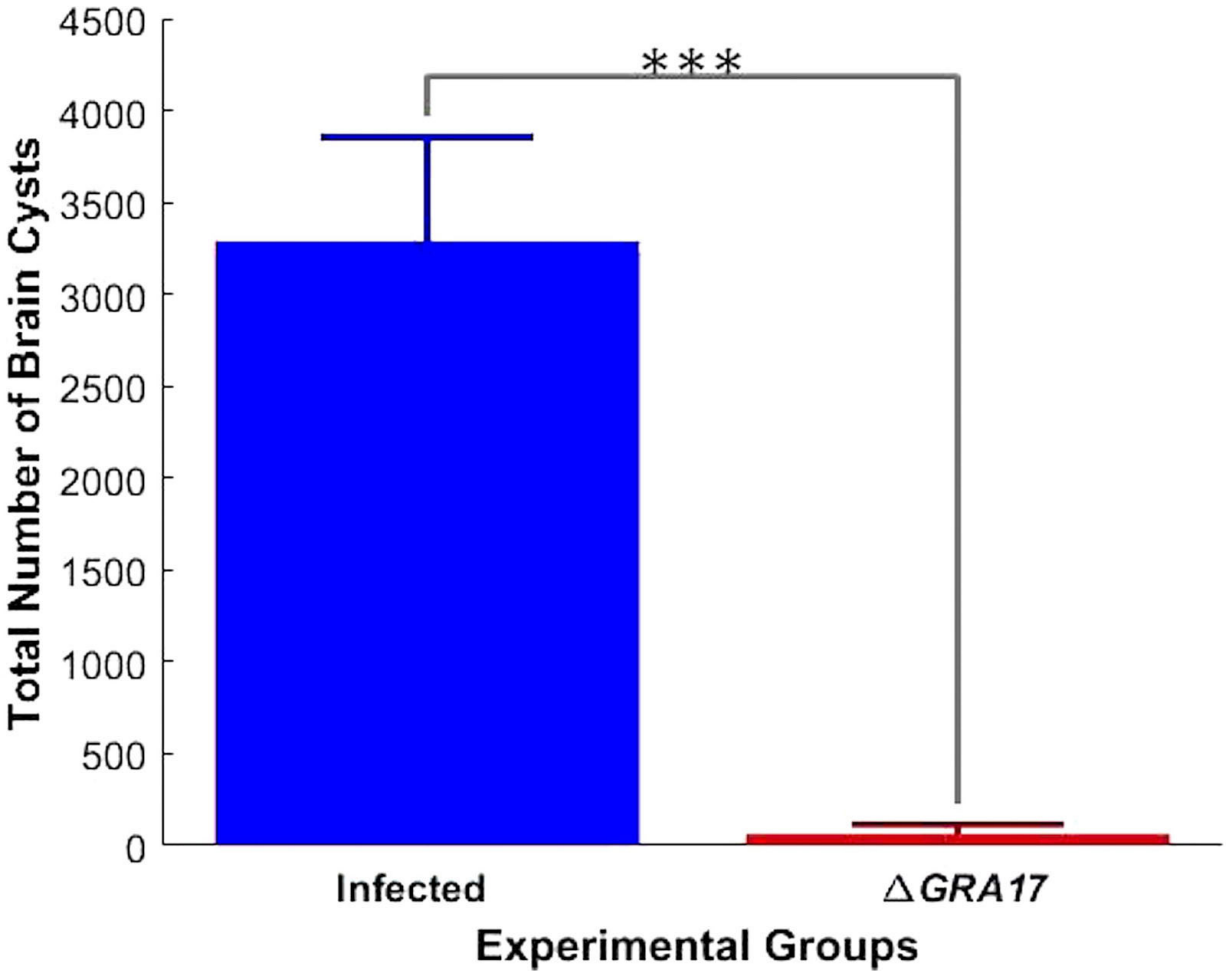
Immunization decreases the parasite tissue cyst burden in infected dams. Female mice were immunized with Δ*GRA17* and 70 days after they were mated and gavaged orally with 10 Pru cysts on day 12 of gestation. At 30 days after delivery, the cysts loads in brain tissue were analyzed. Non-immunized dams showed higher tissue burdens of cysts than immunized mice. Data points indicate means ± SD. Significance compared with infected mice: ***p < 0.001.

**Figure 12:**
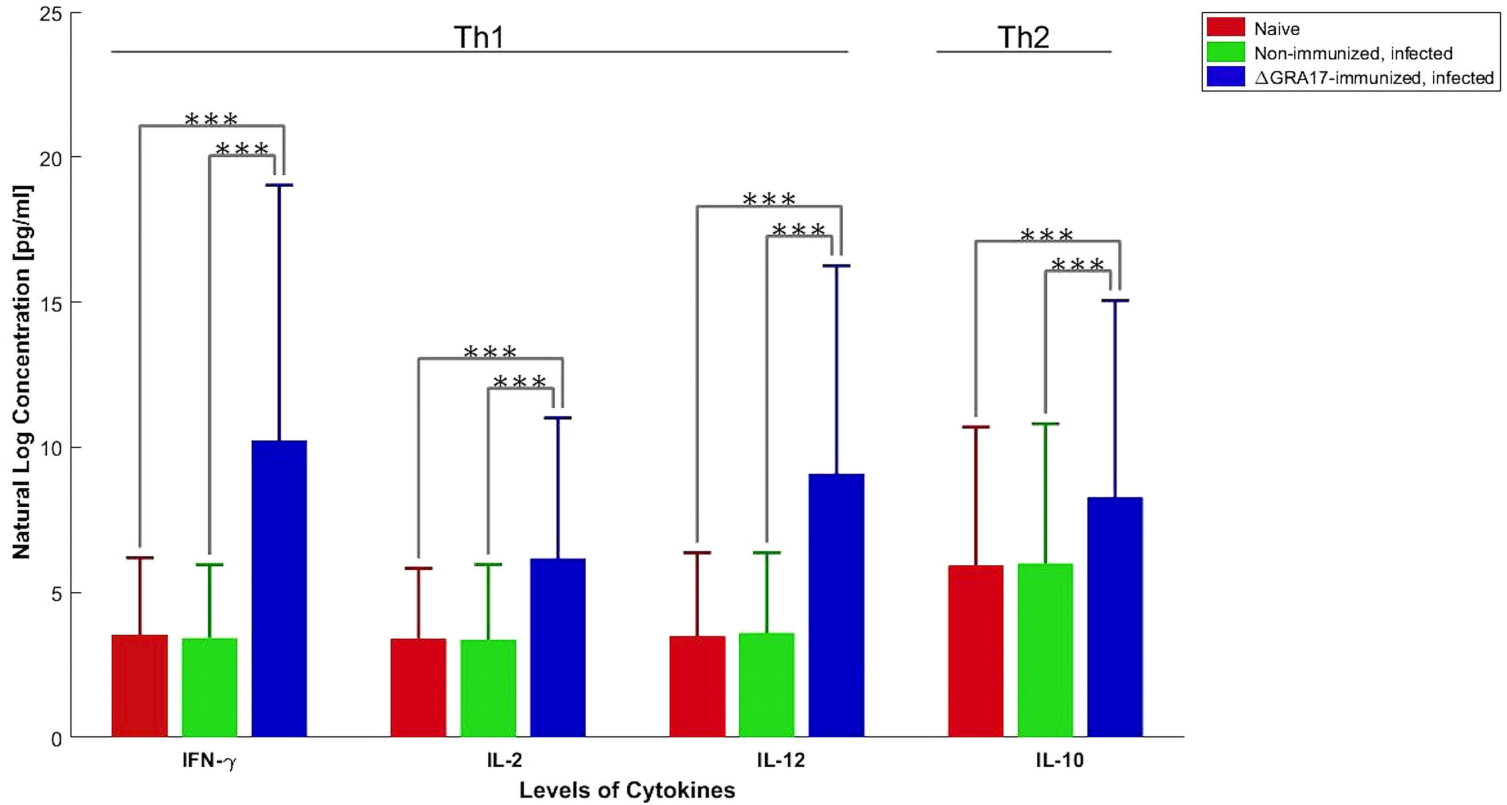
Production of *Toxoplasma gondii*-specific Th1 and Th2 cytokines in *GRA17*-immunized mice after infection with Pru cysts on day 12 of gestation. Immunized, pregnant mice were infected with *T. gondii* Pru cysts 12 days of gestation and spleen cells were collected 6 days after infection. Splenocyte culture was stimulated *in vitro* with *T. gondii* soluble tachyzoite antigen (10 µg/ml). Cell-free supernatants were harvested and evaluated for interleukin-2 (IL-2), IL-10, IL-12, and INF-γ. Cytokine concentrations represent mean ± SD after subtraction of background control values with medium only. Significance compared with uninfected and non-immunized (naïve) mice: ***p < 0.001.

Pregnant dams were infected orally with 10 cysts of *T. gondii* Pru strain on day 12 of gestation. After birth, litter size, survival rate, and BW of pups were determined. As shown in Figure 10A, the number of neonates per litter from immunized and infected mice was significantly higher than that of non-immunized and infected mice (*P* < 0.05), although no significant difference in the litter sizes, and survival rate and BW of pups (35 days after birth) was observed between immunized and infected mice, and non-immunized and uninfected mice. In contrast, survival rate and BW of pups from non-immunized and infected mice were significantly lower than that of non-immunized and uninfected, and immunized and infected mice (Figure 10B). One month and 35 days after birth, dams and their pups were euthanized, respectively, and the number of cysts in their brain tissue was assessed. The tissue cysts were found in all pups born to non-immunized and infected dams, whereas cysts were detected in only 48.2% of pups born to immunized and infected dams. The average number of brain tissue cysts in pups from immunized and infected mice were significantly lower than that of non-immunized and infected dams (56 ± 25 cysts/brain, *n* = 27 vs. 919 ± 339 cysts/brain, *n* = 19; *P* < 0.001). Also, Δ*GRA17*-immunized dams displayed significantly lower parasite burdens in the brain than non-immunized mice (Figure 11). Stimulation of maternal splenocytes with *T. gondii* antigen, 18 days of gestation (pregnant mice were infected with Pru cysts on day 12 of gestation), resulted in the production of higher amounts of *T. gondii*-specific IFN-γ, IL-2, IL-12 (Th1), and IL-10 (Th2) cytokines in immunized and infected mice compared with non-immunized and infected mice or non-immunized and uninfected mice (*P* < 0.001; Figure 12). However, there was no significant difference in levels of these cytokines between non-immunized and infected mice, and non-immunized and uninfected mice (*P* > 0.05). It remains to be elucidated why infection with Pru strain at 12 days of gestation failed to elicit more cytokines than control, uninfected, mice.

## Discussion

Here, we utilized CRISPR-Cas9 method to generate Δ*GRA17* mutants, which had a phenotype similar to that reported previously (24). Next, we investigated the potential of immunization with *GRA17*-defective mutants of *T. gondii* RH strain, as a live-attenuated vaccine, to protect Kunming mice against acute, chronic, and congenital forms of toxoplasmosis. We also quantified the levels of antibodies and cytokines evoked in mice against challenge with Δ*GRA17* mutant using ELISA. The pattern of the immune response observed in immunized mice suggested a sequential Th1 and Th2 T cell response, reflecting the activation of both cell- and antibody-mediated responses.

### Immune Correlates of Protection

A major goal of vaccine development is the induction of adequate immune responses against antigens delivered by the mucosal route. Δ*GRA17*-immunized mice developed a high level of anti-*T. gondii* IgG at 28 and 70 days after immunization. This level of antibodies plays important role in protection against subsequent infection with *T. gondii* tachyzoites and in controlling *T. gondii* during the chronic phase of infection by preventing cysts’ reactivation (34). We next analyzed the antibody types elicited against immunization with Δ*GRA17* mutant by studying the antibody subclasses (IgG1 and IgG2a). Our results showed that immunization with live-attenuated Δ*GRA17* strain promoted both cell- (Th1) and humoral- (Th2) mediated immune responses in Kunming mice. Immunized mice exhibited IgG2a as the predominant antibody’s class during early infection followed by upregulation of IgG1 in the chronic phase, probably as a result of Th1 to Th2 switching. A high ratio of IgG2a to IgG1 antibody titers observed at 28 days after immunization indicated that Th1 immune response was induced. However, at 70 days after immunization the level of IgG1 antibody increased, suggesting the induction of a mixed Th1/Th2 immune response. These results demonstrated a sequential upregulation of antiparasite IgG2a and IgGl antibodies. This isotypes’ pattern is supported by the patterns of cytokines detected in the supernatants from spleen cell cultures where the levels of Th1-type cytokines (IFN-γ, IL-2, and IL-12) and Th2-type cytokine (IL-10) in Δ*GRA17*-immunized mice were significantly higher compared to naïve mice. The fact that IgG2a is known to be elicited by IFN-γ in mice (35) is consistent with our finding where a bias to Th1 response (i.e., higher IgG2a/IgG1) was correlated with early IFN-γ production, pointing to the important role of IFN-γ in eliciting high IgG2a responses against avirulent *T. gondii*, therefore, influencing the immunogenicity of this vaccine.

### Protection against Acute and Chronic Toxoplasmosis

High levels of Th1-type cytokines including IFN-γ, IL-2, and IL-12 developed in immunized mice might have provided immune-protection to mice against challenge with a lethal dose of wt strains (type I RH or two ToxoDB#9 strains). Deaths occurred by days 7–10, with 100% mortality in non-immunized and infected mice, whereas all immunized mice survived. Seven days following a subsequent challenge with *T. gondii* RH strain a significantly higher (*P* < 0.001) level of IFN-γ and IL-12 was detected in the peritoneal washes and sera of non-immunized and infected mice compared to Δ*GRA17*-immunized and infected mice. This finding suggests that the response of the naïve and infected mice was of an inflammatory nature and that Δ*GRA17*-immunized and infected mice developed a balanced pro-(Th1) and anti-inflammatory (Th2) cytokine response. The remarkably high levels of IFN-γ and IL-12 and the associated over-inflammation are probably the basis for adverse health complications developed in naïve and infected mice as previously reported (36, 37). *T. gondii* infection is known to induce a Th1-biased inflammatory response by stimulating T cells, macrophages, natural killer cells, dendritic cells, and neutrophils to produce IFN-γ and IL-12 (38–41). These two cytokines are central to the development of immunity against *T. gondii* infection. Suppression of IL-2 by effector T cells triggered by *T. gondii* infection can cause Treg cells to lose their capacity to balance Th1 immune responses, eventually leads to immunopathogenesis and the death of mice during acute *T. gondii* infection (42). On the other hand, the Th2 cytokine IL-10 is essential for counterbalancing the exacerbated inflammatory Th1 response that induces immunopathologies (36). Therefore, the balanced level of Th1/Th2 cytokines observed in Δ*GRA17*-immunized and infected mice was adequate to control the dissemination of tachyzoites, but not excessive enough to elicit significant inflammation. The immune response triggered by Δ*GRA17* immunization also fully protected mice against oral infection with type II Pru cysts. Our results showed that only 40% non-immunized mice and orally infected 20 Pru cysts survived, whereas all immunized mice (100%) survived. It is worth mentioning that although the number of cysts in immunized mice was significantly reduced, immunization did not prevent the establishment of chronic infection and cyst formation in 80% of the immunized mice.

### Impact on Congenital Toxoplasmosis

We assessed the protection conferred by immunization against transplacental transmission and results showed that immunized mice infected with 200 tachyzoites of *T. gondii* RH strain on day 18 of gestation did not show any significant differences in the litter size, survival rate, or mean BW of pups at day 5 after birth compared to pups from non-immunized and uninfected mice. Also, immunization protected completely the dams form developing clinical signs of toxoplasmosis. In contrast, non-immunized and infected dams developed clinical disease and aborted. Furthermore, the litter size, survival rate, and mean BW of their pups were significantly reduced. The small litter size and low survival rate of pups born to non-immunized dams can be attributed to *T. gondii* infection because parasite DNA was detected in the aborted fetuses. The reduced BWs of pups may be caused by the limited care and postnatal malnutrition as a consequence of infection of the dams.

Likewise, in the immunized pregnant mice orally gavaged with Pru cysts on day 12 of gestation the litter size, survival rate and mean BW of the pups at day 35 after birth were not different from that of non-immunized and uninfected mice. In contrast, the litter size, survival rate, and mean BW of pups from non-immunized and Pru-infected mice were significantly reduced (*P* < 0.05). It can be argued that vaccination in pregnant mice with a live-attenuated parasite strain may be fraught with the possibility of transmission of infection to the fetus. However, although the immune responses triggered by Δ*GRA17* immunization did not completely block the vertical transmission, a significantly less tissue cyst burden was observed in the brain of pups from immunized and Pru-infected mice. As expected, all pups of mock-immunized and Pru-infected dams had more brain cysts. With regard to the brain tissue cyst load in infected dams, there was a significantly larger number of brain cysts in non-immunized than immunized dams. These results indicate that immunization with Δ*GRA17* can induce a partial protective immunity against subsequent i.p. infection with *T. gondii* tachyzoites RH strain during the third trimester of gestation and oral infection with Pru cysts during the second trimester of gestation.

Further analysis of the cytokines secreted by maternal spleen cells of immunized dams revealed higher levels of Th1-type cytokines (IFN-γ, IL-12, and IL-2) and Th2 cytokine (IL-10) in immunized and Pru-infected compared to non-immunized and infected mice, and non-immunized and uninfected mice. This cytokines’ pattern in immunized and infected mice is similar to that observed at 70 days after immunization in non-pregnant mice. But, *T. gondii* is known to stimulate a strong Th1 response characterized by the early classical activation of macrophages and the production of pro-inflammatory mediators, such as IL-12, IFN-γ, and nitric oxide. Th1 response can suppress the development of alternative activation of macrophages and, thus, Th2 responses. Thus, while high level of anti-*T. gondii* Th1 immune response is required to control the materno–fetal transmission of *T. gondii* (43) and prevent congenital transmission (44, 45), it can adversely affect the pregnancy (37).

The increased IL-10 level in our study is interesting because this cytokine plays a critical role in downregulating IFN-γ responses in C57BL/6 mice following *T. gondii* infection. IL-10-deficient C57BL/6 mice displayed elevated IL-12 levels and consequently increased IFN-γ and TNF-α responses, which resulted in hepatic inflammation and necrosis (36). Previous work showed a higher rate of materno–fetal transmission of *T. gondii* in IFN-γ-deficient C57BL/6 mice compared to wt C57BL/6 mice (43). On the other hand, the pregnancy outcome of C57BL/6 mice infected with *T. gondii* improved by treatment with recombinant IL-10 and deteriorated in IL-10-deficient mice compared to *T. gondii*-infected naïve mice (46). Increased level of the Th2-type cytokine, IL-10, restrains Th1 immune response and Th1-mediated immunopathology, while maintaining a Th2 immune response (47, 48).

During pregnancy, the maternal immune response is shifted toward a Th2 cytokine profile in order to accommodate the growing fetus and to ensure maintenance of the pregnancy. Therefore, while the high level of Th1-type cytokines in the immunized mother plays an important role in countering congenital transmission (44, 45), Th2-type cytokine IL-10 is needed to balance Th1 immune response in order to maintain the pregnancy. In agreemnt with these findings, we observed that pregnancy was not successful in mice mated 1 month after immunization where Th1 response was dominating. The role of IL-10 in preventing immunopathology during toxoplasmosis is a topic of great interest. CD4^+^ T lymphocyte cells derived from mice infected with *T. gondii*-irradiated tachyzoites were found to co-produce IFN-γ and IL-10 (49). Also, IL-10 production was found to require a “reactivation” step involving IL-12 production and subsequent IFN-γ production by CD4 T cells (50). Therefore, simultaneous production of antagonistic IL-10 and IFN-γ cytokines by CD4 Th1 cells in response to *T. gondii* suggests that conditions that maintain anti-*T. gondii* Th1 immunity also promote a negative feedback mechanism for limiting Th1 hypersensitivity and inflammatory tissue damage.

## Conclusion

Our study shows the feasibility of using live attenuated Δ*GRA17* mutant RH strain of *T. gondii* for vaccination against *T. gondii* infection. Our results also indicate that i.p. immunization of Kunming mice with Δ*GRA17* mutant RH strain can generate an immune response protective against *T. gondii* infection and disease at a distant site of challenge. Partial protection was achieved against challenge with homologous and heterologous strains of the same and different genotypes. This effect was demonstrated by an improvement in the survival rate and a reduction in the parasite cyst burden in the brain of immunized mice. Δ*GRA17*-immunized mice developed a sequential Th1 and Th2 response as indicated by high levels of Th1 immunity (IFN-γ, IL-2, IL-12, and IgG2a) at 28 day postimmunization and the elevation of Th2 protective immunity (IL-10 and IgG 1) levels at 70 days postimmunization compared to naïve controls. Partial protection against congenital transmission was demonstrated by significant reduction in the cyst burden in the brain of pups and their dams, and improvement in the survival rate and BW of pups born to immunized dams compared to non-immunized dams. Given the potential efficacy of Δ*GRA17* mutant vaccine, the data generated in mice should be extended to higher animal models.

## Author Contributions

X-QZ, S-YH, and HE designed the experiments, interpreted the data, and critically revised the manuscript. J-LW, W-NZ, KC, and T-TL performed the experiments and analyzed the data. J-LW drafted the manuscript. D-MY and X-XZ helped in the implementation of the study. All authors reviewed and approved the final version of the manuscript.

## Conflict of Interest Statement

The authors declare that the research was conducted in the absence of any commercial or financial relationships that could be construed as a potential conflict of interest.

## Supplementary Material

Table S1 Details of single guide RNA and identification primers used in this study.

Figure S1 Schematic illustration of the study design. Experimental overview of the present investigation from immunization of Kunming mice with 5 × 10^4^ Δ*GRA17* tachyzoites **(A)**, assessment of immune responses in the serum of immunized mice prior to infection **(B)** to the evaluation of the efiicacy of the immunization against acute infection **(C)**, chronic infection **(D)**, and congenital infection **(E)**. For simplicity in this illustration, only the experiments that involved the immunized and infected groups are shown. More details about other experimental mouse groups and controls can be found in “Materials and Methods.” Abbreviations: i.p., intraperitoneal; dpi, days post infection.
