# Supplementary material for: Immunization with Toxoplasma gondii GRA17 Deletion Mutant Induces Partial Protection and Survival in Challenged Mice
Source: Front Immunol. 2017 Jun 29;8:730. doi: 10.3389/fimmu.2017.00730 (PMC5489627; doi:10.3389/fimmu.2017.00730)
Supplement: Table S1 — Details of single guide RNA and identification primers used in this study. [file Table_1.DOCX]

**TABLE S1｜The information of sgRNA and identification primers used in this study**

| Gene | Plasmid | sgRNA | KO-GRA17-F | KO-GRA17-R |
| --- | --- | --- | --- | --- |
| GRA17 | pSAG1::CAS9-U6::GRA17 | GACTGTCCCTGAGGACCCAT | CAATCCAGGGACGAACCATT | TCTGCTTCACGGCCATCTT |
