# Supplementary figures and images for: Immunization with Toxoplasma gondii GRA17 Deletion Mutant Induces Partial Protection and Survival in Challenged Mice
Source: Front Immunol. 2017 Jun 29;8:730. doi: 10.3389/fimmu.2017.00730 (PMC5489627; doi:10.3389/fimmu.2017.00730)

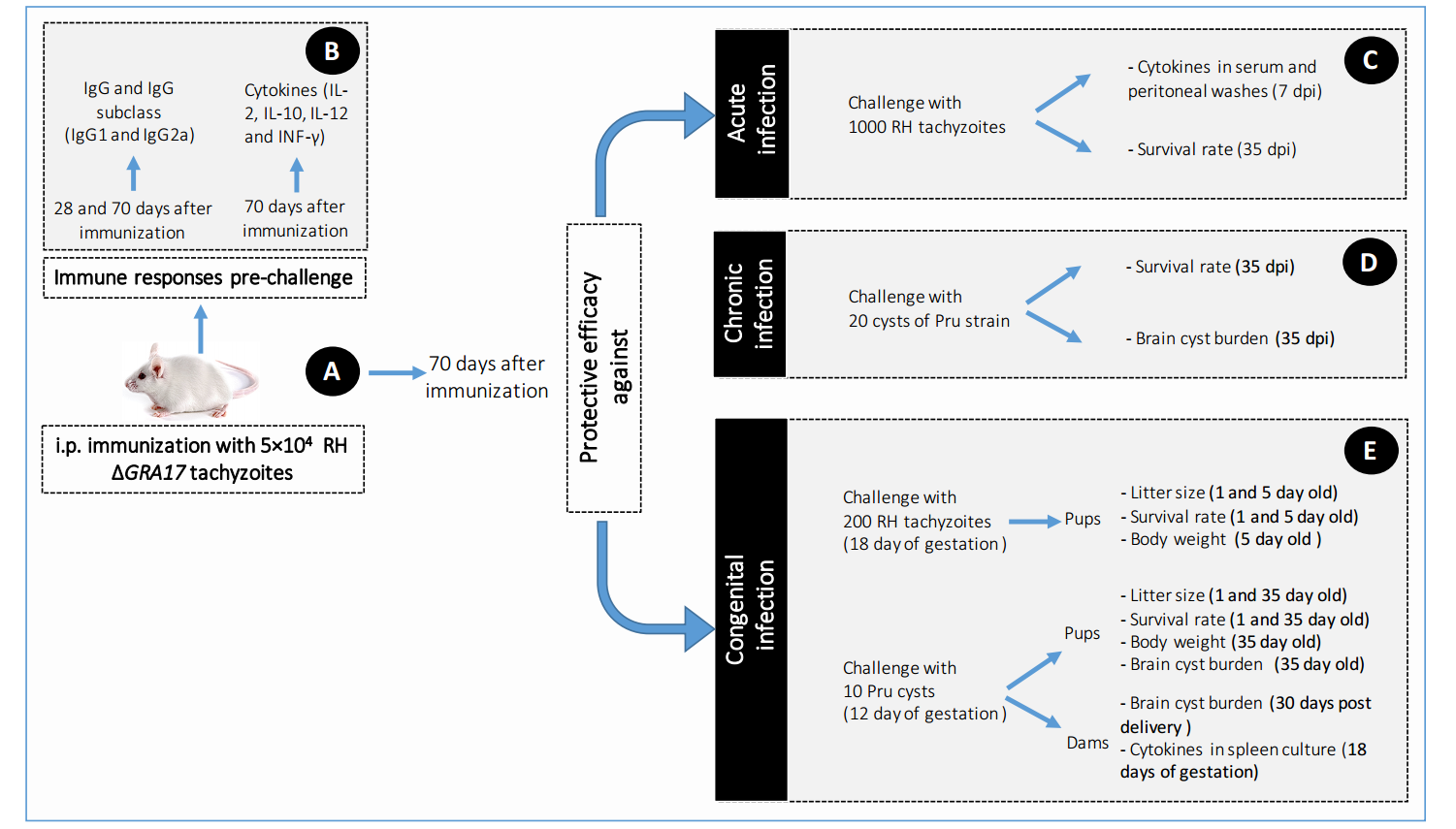

Supplement: Figure S1 — Schematic illustration of the study design. Experimental overview of the present investigation from immunization of Kunming mice with 5 × 10^4^ ΔGRA17 tachyzoites (A), assessment of immune responses in the serum of immunized mice prior to infection (B) to the evaluation of the efiicacy of the immunization against acute infection (C), chronic infection (D), and congenital infection (E). For simplicity in this illustration, only the experiments that involved the immunized and infected groups are shown. More details about other experimental mouse groups and controls can be found in “Materials and Methods.” Abbreviations: i.p., intraperitoneal; dpi, days post infection. [file Image_1.TIF]
